# Inverse association between plasma chlordecone concentrations and progression of alcoholic liver fibrosis: the role of liver metabolism

**DOI:** 10.1186/s12940-024-01054-6

**Published:** 2024-03-20

**Authors:** Moana Gelu-Simeon, Marie-Josée Lafrance, Leah Michineau, Eric Saillard, Jean Pierre Thomé, Claude Emond, Michel Samson, Luc Multigner

**Affiliations:** 1 CHU de la Guadeloupe, Univ Antilles, Inserm, EHESP, IRSET (Institut de recherche en santé, environnement et travail) - UMR_S 1085, Route de Chauvel, Pointe-à-Pitre Cedex, Guadeloupe 97159 France; 2 Service d’Hépato-Gastroentérologie, Centre Hospitalier Universitaire de Guadeloupe, Pointe à Pitre, Guadeloupe France; 3 Univ Rennes, Inserm, EHESP, IRSET (Institut de Recherche en Santé, Environnement et Travail) - UMR_S 1085, Avenue du Professeur Léon Bernard, Rennes, F-35000 France; 4 Université de Liège, LEAE -CART, Freshwater and Oceanic Sciences Unit of Research (FOCUS), B6C, Liège, 4000 Belgium; 5 PKSH Inc, Crabtree, QC Canada; 6 École de Santé Publique, Département de Santé Environnementale et Santé au Travail (DSEST), Université de Montréal, Montreal, QC Canada

**Keywords:** Chlordecone, Persistent organic pollutants, Liver fibrosis, Reverse causality

## Abstract

**Background and Aims:**

Chlordecone is a persistent organochlorinated insecticide, extensively used in the French West Indies and has been contaminating the population for more than thirty years. Its potentiation effect on hepatotoxic agents has been demonstrated in animal models. We investigated the relationship between environmental exposure to chlordecone and the progression of liver fibrosis.

**Methods:**

This study included 182 consecutive patients with chronic alcoholic hepatitis whose liver fibrosis was assessed using non-invasive methods. Measured plasma chlordecone concentrations at inclusion were used as surrogate of long-term exposure under steady-state conditions. As the pharmacokinetic processing of chlordecone is largely determined by the liver, we used a human physiologically based pharmacokinetic model to predict plausible changes in the steady-state blood chlordecone concentrations induced by liver fibrosis.

**Results:**

With a median follow-up of 27.1 years after the onset of alcohol consumption, we found a significant decrease in the risk of advanced liver fibrosis with increasing plasma chlordecone concentration (adjusted hazard ratio = 0.56; 95% confidence interval: 0.34–0.95 for the highest vs. lowest tertile, *p* = 0.04). Changes induced by liver fibrosis influenced the pharmacokinetic processing of chlordecone, resulting in substantial modifications in its steady-state blood concentrations.

**Conclusion:**

According to this human model of coexposure to alcohol, reverse causality is the most plausible explanation of this inverse association between plasma chlordecone concentrations and progression of liver fibrosis. This study underlines the importance of considering the pharmacokinetic of environmental contaminants in epidemiological studies when biomarkers of exposure are used to investigate their own impact on the liver.

**Trial registration:**

ClinicalTrials.gov Identifier: NCT03373396.

**Supplementary Information:**

The online version contains supplementary material available at 10.1186/s12940-024-01054-6.

## Background

There is a growing number of experimental and epidemiological studies suggesting that long-term exposure to man-made environmental chemicals may be involved in the occurrence and progression of liver diseases, such as metabolically associated fatty liver disease, chronic active hepatitis, cirrhosis, and hepatocellular carcinoma [1–4].

Among environmental contaminants, halogenated persistent organic pollutants (POPs) have attracted attention because of their persistence in the environment and bioaccumulation in human beings. In addition, they can interfere with hormone-, glucose-, and lipid-metabolism-regulated processes, for which the liver plays a key role [5–7].

Chlordecone, also known as Kepone, is an organochlorinated pesticide listed in the Stockholm convention on POPs. Experimental studies, in vitro and in vivo, have shown that chlordecone is a neurotoxic, reprotoxic, and carcinogenic substance with well-recognized estrogenic properties [8]. In 1975, a chemical disaster affected chlordecone manufacturing workers in the industrial city of Hopewell (Virginia, US) [9]. Male workers exposed to high amounts of this chemical (blood concentration in the range of mg/L) showed a clinical syndrome characterized by appendicular ataxia and tremors, low sperm-cell counts and mobility, and hepatomegaly with normal liver marker enzyme levels [9, 10]. Analyses of chlordecone in various tissues showed that it preferentially accumulates in the liver, unlike other POPs, for which fatty tissues constitute the main site of accumulation [10]. Light and electronic microscopic examination of biopsies from enlarged livers showed distinct non-specific lesions [11]. Two to three years after this episode of acute exposure, the chlordecone completely cleared from blood, most clinical signs disappeared, and the liver returned to normal size. It was concluded that the hepatic response to the presence of chlordecone represents adaptive changes rather than hepatotoxicity per se [11, 12].

Although it is not hepatotoxic, one intriguing property of chlordecone is its ability to potentiate the liver damage induced well-known hepatotoxic agents. Indeed, experimental studies in rodents have shown that prior exposure to non-toxic levels of chlordecone markedly amplifies the hepatotoxic effects of low single doses of chlorinated and brominated halomethanes [13, 14] and acetaminophen [15]. Recent studies in rodents have shown that pre-exposure to chlordecone also enhances hepatic fibrosis induced by carbon tetrachloride [16] and the progression of hepatitis induced by Concanavalin A and murine hepatitis virus [17]. Such co-exposure was also shown to increase serum transaminase levels and histopathological lesions, such as necrosis, as well as the expression of genes encoding components of the extracellular matrix, and the progression of liver fibrosis [16, 17].

After the US production of chlordecone was discontinued in 1976, the pesticide was produced by a French company and then extensively used to fight banana weevils in the French West Indies (FWI) until 1993 [18]. Because of its extremely low biotic and abiotic degradation, chlordecone is still present in the soil where it was applied and contaminates water resources, as well as the local aquatic and terrestrial food chains. Consequently, human beings continue to be exposed to this chemical by the consumption of contaminated foodstuffs. Epidemiological studies conducted in the FWI have shown that continuous exposure to chlordecone at environmental levels (blood concentration in the range of µg/L) is associated with an increased risk of prostate cancer, preterm birth, and impaired child neurodevelopment [19–21].

We do not know whether chlordecone at environmental exposure levels, as occurs in FWI populations, can potentiate histopathological lesions induced by common hepatotoxic agents. The main objective of this study was to investigate the relationship between chlordecone exposure and the progression of liver fibrosis among patients with active chronic liver disease. We extended this study to two other universally widespread POPs, *p,p’*-dichlorodiphenyldichloroethylene (DDE, the main and most stable metabolite of the insecticide *p,p’*-dichlorodiphenyltrichloroethane, DDT) and the industrial byproduct polychlorinated biphenyl congener 153 (PCB-153). Experimental studies in rodents have shown that these compounds also potentiates the liver damage induced by carbon tetrachloride [22, 23]. Alcohol was used as a model of chronic liver disease associated with coexposure to chlordecone. Chlordecone and other POPs exposure were assessed at the time of the stage of liver fibrosis by measuring their concentration in the blood, a procedure that accurately reflects the long-term body burden of persistent compounds.

The liver is strongly involved in the absorption, distribution, metabolism, and elimination of chlordecone [8]. Consequently, it cannot be excluded that liver fibrosis may induce changes in the steady-state plasma concentration of chlordecone and thus alter the surrogate of exposure. We assessed this issue by simulating potential changes in the steady-state blood chlordecone concentration induced by liver fibrosis using a human physiologically based pharmacokinetic (PBPK) model [24].

## Patients and methods

### Study population

This study was conducted at the University Hospital of Guadeloupe, a French archipelago in the Caribbean of 405,000 inhabitants. Between November 2011 and December 2013, we invited all patients over 18 years of age consulting for active chronic hepatitis of alcoholic etiology, defined by an elevated transaminase (alanine aminotransferase (ALT) and/or aspartate aminotransferase (AST)) above the upper limit of the normal range, and who regularly consumed alcohol, with daily consumption over 20 g/day (women) or 30 g/day (men), to participate in the study [25]. We excluded pregnant women and any patients with hepatocellular carcinoma, autoimmune hepatitis, hemochromatosis, Wilson disease, human immunodeficiency virus infection, or a previous history of liver decompensation. All patients were screened for hepatitis B virus (HBV) and hepatitis C virus (HCV).

At enrolment, patients were interviewed in person to obtain information about their date of birth, year of onset of alcohol consumption, education, current weight and height, weight change during the previous 10 years, smoking, recreational drug use, and coffee consumption, and whether they had diabetes mellitus. Alcohol consumption was self-reported based on the mean number of drinks per day and the volume and category of alcoholic beverages during the previous year and the physician calculated the average daily pure ethanol consumption in grams /day [26]. The body mass index (BMI) was calculated as the weight (kg)/height (m^2^) and categorized as normal (≤ 25), overweight (> 25 and < 30), and obese (≥30). The percentage of weight loss was calculated as (weight 10 years prior minus the current weight) x100 and categorized as < 10 and ≥ 10%. Participants were also asked to provide a blood sample.

The study was approved by the relevant ethics committee for studies involving human subjects (Comité de Protection des Personnes Sud-Ouest et Outremer III, n° 2011-A00124-37). Each participant received, completed, and signed an informed consent form.

### Assessment of liver fibrosis

At enrollment, all patients underwent assessment of liver fibrosis by two non-invasive methods, one liver stiffness measurement and one blood-based test. Liver stiffness was measured by transient ultrasound elastography (Fibro Scan®, EchoSens, Paris) [27] and the results from the FibroTest® (Biopredictive, Paris) [28] or Fibrometer Alcohol® [29] blood-based tests were used as surrogate serum biomarkers combined into composite scores. The thresholds used for staging liver fibrosis according to each non-invasive tool are presented in Table S1. In the event of discrepancies between elastography and the blood-based tests, a liver biopsy was performed. Liver fibrosis was staged according to the METAVIR scoring system [30] on a scale from F0 to F4; F0 = no fibrosis, F1 = minimal fibrosis without septa, F2 = septal fibrosis with a few septa, F3 = severe fibrosis with numerous septa, and F4 = cirrhosis.

### Chlordecone and other POPs measurements

Chlordecone, DDE, and PCB-153 were measured in the plasma fraction of blood samples provided at enrolment and were analyzed by high-resolution gas chromatography using an instrument (Thermo Quest Trace 2000, Milan, Italy) equipped with a Ni-63 electron capture detection system. Detailed information about the sampling, analysis, and quality assurance and controls have been published elsewhere [19, 31]. The analytical limit of detection (LOD) was 0.02 mg/L for chlordecone and 0.05 µg/L for DDE and PCB-153.

### Biochemical analyses

Plasma concentrations of albumin, total and conjugated bilirubin, total cholesterol, and total triglycerides were recorded at the time of the assessment of liver fibrosis. The total lipid concentration was calculated as previously described [32].

### Chlordecone pharmacokinetic simulations

Following oral exposure, chlordecone is quickly and largely absorbed (more than 90%) by the intestinal wall, its high bio-accessibility being made possible by the solubilizing action of bile salts [8]. It is then distributed to various tissues, with the highest concentrations found in the liver [8]. Chlordecone in the blood is always found as the parent compound, carried by albumin and high-density lipoproteins (HDL) [33], which are associated with reverse cholesterol transport pathways [34]. In the liver, chlordecone is firmly bound to cytosolic proteins called chlordecone-binding proteins (CDBPs) [35], promoting its sequestration or partially reducing it into chlordecone-alcohol by chlordecone reductase, an aldo-keto reductase also named AKR1C4 [36, 37] Chlordecone-alcohol and its glucuronide conjugate are then excreted into the bile and eliminated through the feces [36] However, more than 90% is removed from the bile in the intestine and undergoes enterohepatic recirculation after deconjugation and oxidation in the intestinal lumen [10] Such enterohepatic recirculation explains the long elimination half-life of chlordecone of 131 days [24].

For this study, a human chlordecone PBPK model [24] was used to predict steady-state plasma concentrations. The model considered oral exposure, given that contaminated foodstuffs are the only source of chlordecone exposure for FWI populations. Simulations were performed based on the following scenario: 74 kg body weight (bw) with a steady-state plasma chlordecone concentration corresponding to 1 µg/L, resulting in an estimated daily oral exposure of 1.68 × 10^− 2^ µg of chlordecone/kg bw by the PBPK model. To simulate changes in the steady-state blood chlordecone concentration induced by liver fibrosis, we modified the following physiological parameters used in the model: the fraction of blood chlordecone binding to plasma albumin, the chlordecone bile excretion constant, expressed as a first order elimination rate (L/day), the concentration of CDBPs in liver (nmoL/L), the clearance capacity of chlordecone reductase (L/day), and the bio-accessibility, expressed as the percentage of the daily oral dose available.

### Data and statistical analysis

Continuous variables are described as means and percentiles. POP concentrations were categorical as tertiles (based on their distribution in the total population) or continuous variables after log10 transformation. Plasma levels below the LOD were imputed by a maximum likelihood estimation method [38]. Analysis of covariance was used to compare POP concentrations according to the liver fibrosis status and adjusted for covariates associated with both POPs concentrations (Tables S2 and S3) and fibrosis status (Table S4) with p ≤ 0.20. Multivariable Cox proportional hazards regression models and 95% confidence intervals (CIs) were used to estimate the hazard ratio (HR) of fibrosis progression according to the tertiles of plasma organochlorine concentrations. Plasma levels equal to or below the LOD were included in the first (lowest) tertile. The time to event was defined as the duration between the date of the onset of alcohol consumption and the stage of fibrosis that defined the outcome event. Potential confounders were included as covariates in statistical models if they predicted both plasma POPs concentrations (Tables S2 and S3) and fibrosis status (Table S4) with p ≤ 0.20. For each case of exposure, we also considered other POPs as potential confounders, even if chlordecone concentrations have been shown to poorly correlate with DDE and PCB 153 levels (Table S3). The proportional hazards assumption was verified by the log-negative-log survival distribution function of all variables. Tests for linear trends across the categories of POPs plasma levels were performed, with the organochlorine concentration treated as a continuous variable. Statistical analyses were carried out using MedCalc software version 20.111 (MedCalc Software Ltd, Ostend, Belgium). All tests were two-sided, and p ≤ 0.05 was considered statistically significant.

## Results

The results presented here were obtained from an initial study population of 182 patients presenting with active alcoholic chronic hepatitis (median daily consumption of 165 g of alcohol; 90.1% of patients with a daily consumption of at least 50 g). During 5,218 person-years of retrospective follow-up after the onset of alcohol consumption, 112 (61.5%) reached the F4 stage of liver fibrosis. The mean retrospective follow-up time was 27.1 years. The baseline characteristics of the study population are shown in Table 1. The detected levels and plasma concentrations of POPs are presented in Table 2. Because of the small number of patients with liver fibrosis stages between F1 and F3, further analyses were restricted to patients with the stage F0 and F4 liver fibrosis.

**Table 1:** Baseline characteristics of the study population

Characteristics	N (%)
Number of patients	182
Age at enrolment (mean, SD)	53.7 (11.6)
Sex	
Men	162 (89.0)
Women	20 (11.0)
Education	
Primary	42 (23.1)
Secondary	117 (64.3)
High School and higher	23 (12.6)
Body mass Index, kg/m^2^	
Normal (< 25)	139 (76.4)
Overweight (25 – <30)	37 (20.3)
Obesity (≥ 30)	6 (3.3)
Weight loss > 10% during the last 10 years	
No	143 (78.6)
Yes	39 (21,4)
Alcohol consumption *, g/day (median)	165
Alcohol consumption *, g/day	
< 50	18 (9.9)
≥ 50	164 (90,1)
Age at alcohol onset, years	
< 40	167 (91.8)
> 40	15 (8.2)
Smoking	
Never	97 (53.3)
Ever	85 (46.7)
Coffee consumption	
Never	102 (56.0)
Ever	88 (44.0)
Recreational drugs	
Never	169 (92.9)
Ever	13 (7.1)
Diabetes type 2	
No	160 (87.9)
Yes	22 (12.1)
HBV coinfection	
No	174 (95.6)
Yes	8 (4.4)
HCV coinfection	
No	169 (92.9)
Yes	13 (7.1)
Fibrosis stage	
No fibrosis (F0)	45 (24.7)
Fibrosis without septa (F1)	8 (4.4)
Few septa (F2)	11 (6.0)
Many septa (F3)	6 (3.3)
Cirrhosis (F4)	112 (61.5)
Follow-up time, years (mean, SD)	27.1 (13.4)

^*^As pure ethanol equivalent

**Table 2:** Detection and concentrations of persistent organic pollutants in plasma samples (µg/L) from the study population

POPs *	Detection frequency (%)	Percentiles					Max
		10th	25th	50th	75th	90th	
Chlordecone	90.0	0.02	0.05	0.17	0.58	1.15	10.4
DDE	99.4	0.16	0.46	1.08	2.23	4.43	22.2
PCB153	98.3	0.15	0.26	0.51	0.88	1.63	5.68

^*^Chlordecone was measured in 181 plasma samples, and DDE and PCB153 in 179 plasma samples

Univariate analysis of the risk factors for the progression of liver fibrosis from stage F0 to F4 is presented in Table S4. The age at enrolment, a high level of education, the age (> 40 years) at onset of alcohol consumption, smoking, and diabetes mellitus were associated with a high risk of progression of liver fibrosis. By contrast, coffee consumption tended to be associated with a low risk of progression.

Plasma chlordecone concentrations were lower for patients with stage F4 liver fibrosis than those with no fibrosis and the difference remained after adjustment (Table 3). By contrast, plasma DDE concentrations were higher for patients with stage F4 liver fibrosis than those with no fibrosis but after adjustment, the difference was no longer significant. There were no differences in plasma PCB153 concentrations according to the stage of liver fibrosis of the patients, either in the unadjusted or adjusted models.

**Table 3:** Plasma persistent organic pollutant concentrations (µg/L) according to the stage of liver fibrosis

POPs	Unadjusted mean * (CI 95%)		Adjusted mean ** (CI 95%)	
		*P*		*P*
Chlordecone				
No fibrosis (F0)	0.25 (0.15–0.46)	0.03	0.25 (0.14–0.46)	0.04
Cirrhosis (F4)	0.11 (0.08–0.16)		0.11 (0.08–0.17)	
DDE				
No fibrosis (F0)	0.65 (0.39–0.98)	0.04	0.86 (0.58–1.36)	0.48
Cirrhosis (F4)	0.95 (0.82–1.34)		0.97 (0.78 – 1.21)	
PCB153				
No fibrosis (F0)	0.39 (0.28–0.53)	0.19	0.46 (0.34 – 0.61)	0.88
Cirrhosis (F4)	0.49 (0.41–0.60)		0.47( 0.39 – 0.56)	

^*^Back-transformed log value. ^**^For chlordecone: adjusted to gender, coffee consumption, recreational drugs, and total plasma lipids; For DDE: adjusted to age at enrolment, gender, education, age at alcohol onset, weight loss, smoking, and total plasma lipids; For PCB153: adjusted to age at enrolment, education, smoking, and total plasma lipids

Crude and adjusted Cox analysis showed that patients in the second and third tertiles of plasma chlordecone concentration had a significantly lower risk of fibrosis progression than those in the lowest tertile (Table 4). We obtained comparable results when plasma DDE and PCB-153 were additionally included in the multivariable model. The linear relationship between plasma chlordecone concentrations and fibrosis was significant in the unadjusted and in both adjusted models (p-trend = 0.008, 0.04 and 0.05, respectively). For DDE, there was no association, regardless of the model used (Table 4). However, for PCB-153, we observed a lower risk of fibrosis progression for patients in the second and third tertiles of plasma levels than those in the lowest tertile, but this difference was no longer observed in adjusted models (Table 4).

**Table 4:** HR (95% CIs) of the progression of liver fibrosis according to tertiles of plasma chlordecone, DDE, and PCB-153 concentrations

POPs	F0 N (%)	F4 N (%)	Crude HR (95% CI)		Adjusted HR (95% CI)^*^		Adjusted HR (95% CI)^**^	
Chlordecone (µg/L)								
≤ 0.07	7 (15.6)	44 (39.6)	1.0 (reference)		1.0 (reference)		1.0 (reference)	
> 0.07-<0.45	21 (46.7)	31 (27.9)	0.57 (0.33–0.97)	0.04	0.58 (0.34–1.02)	0.06	0.57 (0.33–1.00)	0.05
≥ 0.45	17 (37.8)	36 (32.4)	0.49 (0.30–0.81)	0.005	0.56 (0.34–0.95)	0.03	0.58 (0.35–0.99)	0.05
*p* Trend			0.008		0.04		0.05	
DDE (µg/L)								
≤ 0.64	21 (46.7)	32 (28.8)	1.0 (reference)		1.0 (reference)		1.0 (reference)	
> 0.64-<1.62	13 (28.9)	39 (35.1)	1.00 (0.59–1.72)	0.98	1.27 (0.73–2.24)	0.39	1.22 (0.69–2.18)	0.49
≥ 1.62	11 (24.4)	40 (36.0)	0.76 (0.43–1.35)	0.35	1.05 (0.56–1.95)	0.88	1.05 (0.55–1.99)	0.89
*p* Trend			0.10		0.66		0.83	
PCB-153 (µg/L)								
≤ 0.31	19 (42.2)	36 (32.4)	1.0 (reference)		1.0 (reference)		1.0 (reference)	
> 0.31-<0.71	14 (31.1)	35 (31.5)	0.57 (0.33–0.97)	0.04	0.78 (0.45–1.35)	0.38	0.73 (0.42–1.29)	0.28
≥ 0.71	12 (26.7)	40 (36.0)	0.43 (0.25–0.74)	0.003	0.78 (0.44–1.38)	0.39	0.68 (0.35–1.32)	0.25
*p* Trend			0.02		0.55		0.41	

^*^For chlordecone: adjusted to coffee consumption, recreational drugs; For DDE: adjusted to education, tobacco consumption, age at alcohol onset, age at enrolment; For PCB153: adjusted to education, tobacco consumption, age at enrolment. ^**^For chlordecone: adjusted to coffee consumption, recreational drugs, DDE, PCB-153; For DDE: adjusted to education, tobacco consumption, age at alcohol onset, age at enrolment, chlordecone, PCB-153; For PCB-153: adjusted to education, tobacco consumption, age at enrolment, chlordecone, DDE

**Fig. 1:**
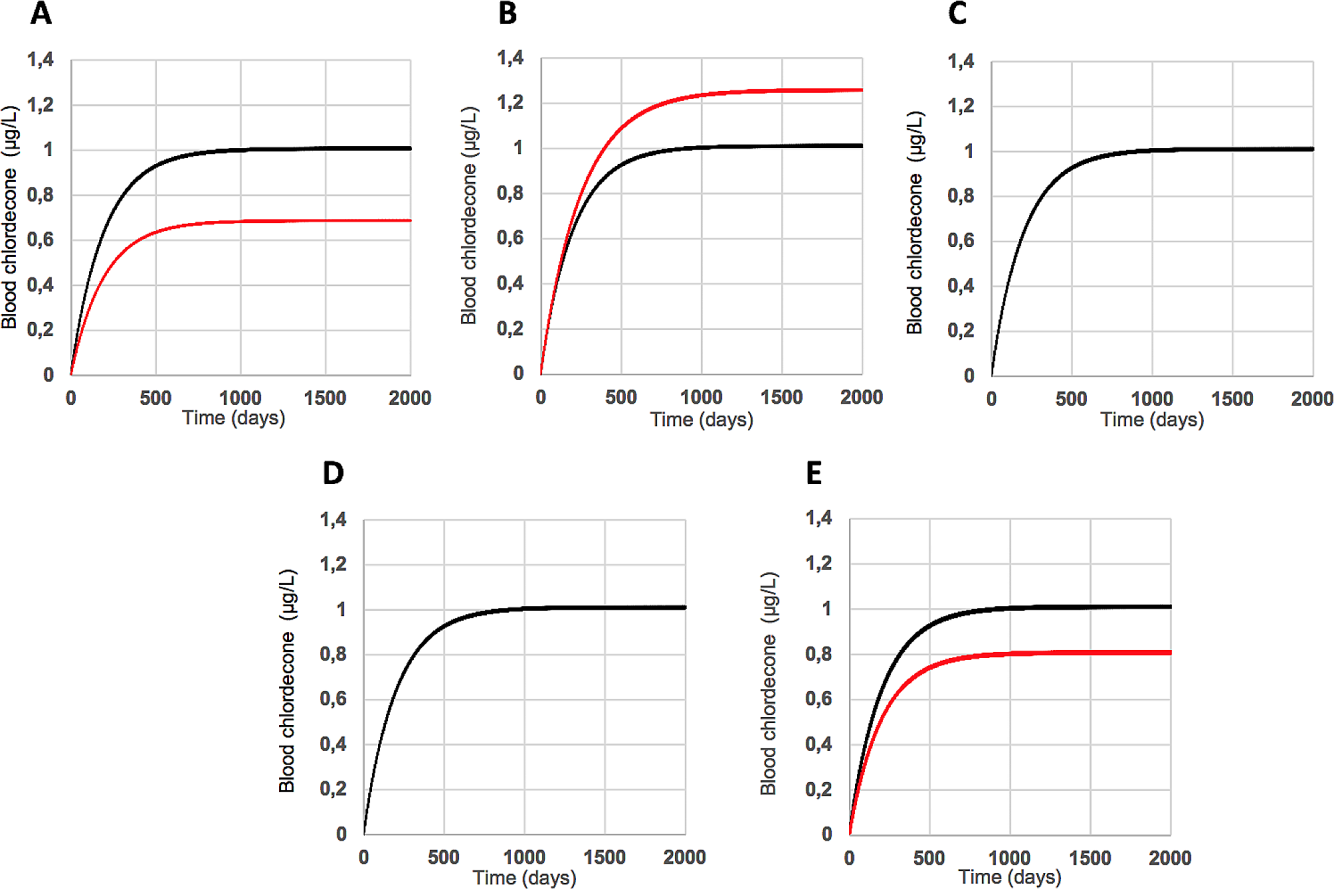
Concentration profiles of chlordecone in the blood (expressed in µg/L of plasma) for chronic daily exposure to 1.68 × 10^− 2^ µg of chlordecone/kg bw (black lines) and for a 20% decrease in the fraction of blood chlordecone binding to albumin (red line, **A**), chlordecone bile excretion (red line, **B**), liver CDBP concentration (the red line is masked by the black line, **C**), clearance capacity of chlordecone reductase (the red line is masked by the black line, **D**), and bio-accessibility (red line, **E**)

The changes in the blood chlordecone concentration profile induced by liver fibrosis are presented in Fig. 1. Based on clinical laboratory analyses (Table S5), which showed 20% lower plasma albumin concentrations for patients with stage F4 liver fibrosis than those with no fibrosis, a 20% decrease in the fraction of blood chlordecone binding to plasma albumin was applied. This resulted in a 32% reduction in the steady-state plasma chlordecone concentration (Fig. 1A).

Significantly higher plasma conjugated bilirubinemia for patients with stage F4 liver fibrosis than those with no fibrosis (Table S5) suggests a decrease in bile flow caused by cirrhosis. It is impossible to correlate an increase in a given plasma conjugated bilirubinemia level to a reduction in the rate of bile flow. Thus, we chose a default decrease of 20% of chlordecone bile excretion, resulting in a 25% increase in the steady-state plasma chlordecone concentration (Fig. 1B).

There is no available data in the literature concerning a decrease in liver CDBPs or chlordecone reductase resulting from liver fibrosis. By default, we chose a decrease in the CDBP concentration of 20% in the liver and 20% in the clearance capacity of chlordecone reductase. Such decreases did not influence the steady-state plasma chlordecone concentration (Fig. 1C and D).

Finally, assuming a default reduction of 20% of bile flow, we simulated a 20% decrease in gut chlordecone bio-accessibility by decreasing the daily oral dose of chlordecone by 20%. This led to a 20% decrease in the steady-state plasma blood chlordecone concentration (Fig. 1E).

## Discussion

This is the first study to investigate the association of the plasma concentrations of chlordecone and other POPs with the progression of liver fibrosis among a cohort of patients with active chronic hepatitis of alcoholic etiology.

We found the adjusted mean plasma concentrations of chlordecone to be lower in patients with advanced stage F4 liver fibrosis than those with no fibrosis and plasma chlordecone concentrations to be inversely associated with the risk of progression of liver fibrosis. By contrast, we found no differences between the adjusted mean plasma concentrations of DDE or PCB-153 and liver fibrosis stage, as well no association between plasma levels of these pollutants and the progression of liver fibrosis.

In this study, patients were consecutively enrolled and liver fibrosis assessed by agreement between several reliable non-invasive methods. The evaluation of alcohol consumption during the last year, as well the year of onset of alcohol consumption, were carefully assessed using standard methods based on self-reporting. We have chosen a model of alcoholic liver disease because of its particularly high representation in our population. This model could be probably generalized to all other etiologies of chronic liver disease, as the chlordecone acts as a potential co-hepatotoxic agent. Exposure to POPs was assessed on the basis of objective determinations of plasma concentrations. This approach covers all exposure routes and, because of the long half-life of POPs in the blood and constant dietary exposure, their plasma concentrations represent a good surrogate of the body burden at steady state, providing a confident estimation of exposure over an extended period. We identified several well-known risk factors (age at onset of alcohol consumption, smoking, type 2 diabetes) and protective factors (coffee consumption) of liver fibrosis progression [39–41]. This lends credibility to the observed inverse association between chlordecone plasma concentrations and liver fibrosis, even if we cannot exclude the presence of unknown or unmeasured confounders. However, such direction of association was unexpected, insofar as it runs counter to our original assumption of a potentiating effect of chlordecone in the progression of liver fibrosis. With the present state of knowledge, there is no observed or experimental data that could confer biological plausibility to a causal relationship between chlordecone exposure and slowing of the progression of liver fibrosis. Therefore, it is essential to determine whether liver fibrosis is likely to interfere with chlordecone pharmacokinetics and thus, question the relevance of plasma chlordecone concentrations as a surrogate of exposure.

We attempted to resolve this issue using a mathematical PBPK model developed for chlordecone that incorporates information about the intrinsic properties and system biology related to the adsorption, metabolism, distribution, and elimination of the substance of interest [42].

Using a forward dosimetry approach, we simulated plasma chlordecone concentrations (internal dose) under the assumption of constant oral daily exposure (external dose) for patients with advanced liver fibrosis (stage F4) or without liver fibrosis (stage F0). Indeed, simulation of a decrease in the fraction of blood chlordecone binding to albumin (related to the observed lower plasma albumin concentration for patients with stage F4 liver fibrosis than those with no fibrosis) resulted in a decreased steady-state plasma chlordecone concentration. By contrast, simulation of a decrease in the storage capacity of chlordecone by the liver or a decrease in the conversion of chlordecone into chlordecone-alcohol by chlordecone reductase did not lead to any change in the steady-state plasma chlordecone concentration. This is not surprising, given the background level of chlordecone exposure of the FWI population, which is insufficient to saturate the CDBPs in the liver or the capacity of hepatic metabolic enzymes unless most of the liver parenchyma is no longer functional. Simulation of a decrease in bile flow, as expected in liver fibrosis and supported by the observed higher blood conjugated bilirubin levels in patients with stage F4 liver fibrosis than those of patients with no fibrosis, resulted in an increased steady-state plasma chlordecone concentration. Finally, simulation of decreased gut bio-accessibility, as a consequence of decreased bile flow and bile acid secretion, resulted in a decreased steady-state plasma chlordecone concentration. A non-biliary mechanism of chlordecone excretion in the gut has been reported when bile is entirely derived through a bile duct T-tube [43]. Under these conditions, chlordecone (as the parent compound) was found in the stool at levels even higher than those observed without biliary diversion. Whether or not a partial reduction in bile flow, as seen for patients with advanced liver fibrosis, results in increased stool excretion by such a non-biliary mechanism is unknown. However, if this is the case, such a process should help to increase the rate of chlordecone removal from the body.

Overall, the magnitude and direction of these simulations, when combined, on mean steady-state blood chlordecone concentrations is not clear. Indeed, except for simulations concerning changes in plasma albumin concentrations, which were based on clinical observations, the tested physiological changes were based on default values because the true values are unknown in our population with liver fibrosis. However, these simulations show that the changes in physiological parameters induced by liver fibrosis could affect the pharmacokinetics of chlordecone by modifying steady-state plasma concentrations while dietary exposure remains constant. Therefore, in the absence of biological plausibility, the reality of the inverse association between plasma chlordecone concentrations and the risk of the progression of liver fibrosis may be questionable and possibly explained by reverse causality.

DDT, the parent molecule of DDE, and a mixture of PCBs have also been reported to potentiate carbon tetrachloride hepatic toxicity in rodents [22, 23] However, we did not observe any association between plasma concentrations of these pollutants and the progression of liver fibrosis. Although the liver is the primary site of metabolic transformation of DDT/DDE and PCBs, unlike chlordecone, they mainly accumulate in adipose tissues and their excretion is largely via the urine [44, 45] Indeed, they are transported in the blood and lymph by LDL and VHDL lipoproteins, which are associated with the centrifugal transport of lipids from the liver to peripheral tissues [34]. Thus, it is possible that liver fibrosis does not lead to significant changes in the pharmacokinetics of these contaminants. However, in the absence of verification using appropriate PBPK modelling simulations, it is difficult to draw any definitive conclusions.

Our pharmacokinetic simulations call into question the validity of plasma chlordecone measurements as surrogate of exposure for patients with liver fibrosis. Thus, it is not possible to draw a conclusion, one way or another, on the true direction of the association, if it exists. Only prospective epidemiological studies in which chlordecone plasma concentrations are estimated before exposure to any chemical or viral hepatotoxic agent will make it possible to verify whether chlordecone exposure potentiates the liver effects induced by hepatotoxic agents.

In conclusion, biomonitoring is a very useful tool for assessing exposure to persistent environmental contaminants. However, because of the key influence of the liver in the control of the pharmacokinetic parameters of these contaminants, the use of exposure biomarkers should be rigorously estimated to prevent erroneous conclusions when they are used in epidemiological studies on chronic liver diseases.

### Electronic supplementary material

Below is the link to the electronic supplementary material.

Supplementary Material 1

## Data Availability

Data are available upon request.

## Abbreviations

POPs: persistent organic pollutants
FWI: French West Indies
PBPK: physiologically based pharmacokinetic
ALT: alanine aminotransferase
AST: aspartate aminotransferase
HBV: hepatitis B virus
HCV: hepatitis C virus
DDE: *p,p’*dichlorodiphenyldichloroethylene
DDT: *p,p’*dichlorodiphenyltrichloroethane
PCB153: polychlorinated biphenyl congener 153
LOD: limit of detection
HDL: high-density lipoproteins
CDBPs: chlordecone-binding proteins
bw: body weight
Cis: confidence intervals
HR: hazard ratio
