# Supplementary material for: Inverse association between plasma chlordecone concentrations and progression of alcoholic liver fibrosis: the role of liver metabolism
Source: Environ Health. 2024 Mar 20;23:30. doi: 10.1186/s12940-024-01054-6 (PMC10953091; doi:10.1186/s12940-024-01054-6)
Supplement: Supplementary file 1 — Supplementary Material 1 [file 12940_2024_1054_MOESM1_ESM.docx]

**Supplemental Tables**

**Inverse association between plasma chlordecone concentrations and progression of alcoholic liver fibrosis: the role of liver metabolism**

**Moana Gelu-Siméon, Marie-Josée Lafrance, Leah Michineau, Eric Saillard,**

**Jean Pierre Thomé, Claude Emond, Michel Samson and Luc Multigner**

**Contents**

**Table S1: Thresholds of noninvasive tests for liver fibrosis assessment in alcoholic liver disease**

**Table S2: Relationships between persistent organic pollutants concentrations (µg/L) and characteristics (as categorized values) of patients**

**Table S3: Correlation between persistent organic pollutants concentrations and characteristics (as continuous values) of patients**

**Table S4: Univariate analysis of risk factors for the progression of liver fibrosis**

**Table S5: Plasma albumin and total and conjugated bilirubin concentrations according to the stage of liver fibrosis**

**Table S1**

**Thresholds of noninvasive tests for liver fibrosis assessment in alcoholic liver disease**

| **Test** | **F0** | **F1** | **F2** | **F3** | **F4** |
| --- | --- | --- | --- | --- | --- |
| FibroTest ^1^ | < 0.29 | 0.29 - <0.49 | 0.49 - <0.59 | 0.59 – <0.88 | ≥0.88 |
| Alcohol Fibrometer ^2^ | <0.18 | 0.18 - <0.59 | ≥0.59 | ND | ≥0.95 |
| Elastography (KPa) ^3^ | <7 | 7 - <8.8 | 8.8 - <12.3 | 12.3 - <19 | ≥19 |

ND : not determined

**Supplemental References**

1. Naveau S, et al. Biomarkers for the prediction of liver fibrosis in patients with chronic alcoholic liver disease. Clin Gastroenterol Hepatol 2005 3:167-174.
2. Calès P, et al. A novel panel of blood markers to assess the degree of liver fibrosis. Hepatology 2005 42:1373–1381.
3. Sandrin L, et al. Transient elastography: a new noninvasive method for assessment of hepatic fibrosis. Ultrasound Med Biol. 2003 29:1705-1713

**Table S2**

**Relationships between persistent organic pollutants concentrations (µg/L) and characteristics (as categorized values) of patients**

| **Characteristic** | **Chlordecone** |  | **DDE** |  | **PCB-153** |  |
| --- | --- | --- | --- | --- | --- | --- |
|  | **Mean ^*^ (CI 95%)** | ***P*** | **Mean ^*^**  **(CI 95%)** | ***P*** | **Mean ^*^**  **(CI 95%)** | ***P*** |
| Sex  Men  Women | 0.16 (0.12 – 0.22)  0.05 (0.02 – 0.13) | 0.02 | 0.81 (0.66 – 0.99)  2.53 (1.48 – 4.33) | 0.002 | 0.46 (0.40 – 0.54)  0.49 (0.23 – 1.07) | 0.80 |
| Education  Primary  Secondary  High School and higher | 0.18 (0.10 – 0.32)  0.14 (0.10 – 0.21)  0.07 (0.03 – 0.21) | 0.27 | 1.52 (0.99 – 2.36)  0.80 (0.64 – 1.00)  0.73 (0.41 – 1.32) | 0.02 | 0.62 (0.45 – 0.87)  0.44 (0.37 – 0.53)  0.36 (0.20 – 0.67) | 0.10 |
| Body Mass Index, kg/m^2^  Normal (< 25)  Overweight (25-<30)  Obesity (>30) | 0.16 (0.11 – 0.22)  0.10 (0.05 – 0.21)  0.07 (0.01 – 1.15) | 0.29 | 0.88 (0.70 – 1.11)  1.05 (0.72 – 1.52)  1.05 (0.13 – 8.45) | 0.76 | 0.46 (0.38 – 0.56)  0.47 (0.36 – 0.60)  0.57 (0.18 – 1.81) | 0.90 |
| Weight loss > 10 % during the last 10 years  No  Yes | 0.13 (0.09 – 0.19)  0.17 (0.09 – 0.34) | 0.49 | 0.85 (0.68 – 1.07)  1.22 (0.84 – 1.78) | 0.14 | 0.45 (0.37 – 0.53)  0.55 (0.38 – 0.79) | 0.29 |
| Smoking  Never  Ever | 0.13 (0.08 – 0.20)  0.15 (0.10 – 0.23) | 0.28 | 1.19 (0.92 – 1.55)  0.68 (0.52 - 0.90) | 0.004 | 0.52 (0.41 – 0.65)  0.41 (0.33 - 0.51) | 0.17 |
| Coffee consumption  Never  Ever | 0.12 (0.08 – 0.17)  0.18 (0.11 – 0.29) | 0.18 | 0.93 (0.74 – 1.17)  0.81 (0.65 – 1.27) | 0.94 | 0.46 (0.37 – 0.57)  0.48 (0.38 – 0.60) | 0.80 |
| Recreational drugs  Never  Ever | 0.14 (0.10 – 0.19)  0.13 (0.04 – 0.43) | 0.90 | 0.95 (0.77 – 1.17)  0.71 (0.39 – 1.27) | 0.34 | 0.47 (0.40 – 0.56)  0.42 (0.27 – 0.63) | 0.61 |
| Diabetes Mellitus  No  Yes | 0.14 (0.10 – 0.19)  0.13 (0.04 – 0.43) | 0.90 | 0.95 (0.77 – 1.17)  0.71 (0.39 – 1.27) | 0.34 | 0.47 (0.40 – 0.56)  0.42 (0.27 – 0.63) | 0.61 |
| Age at alcohol onset, years  < 40  > 40 | 0.15 (0.11 – 0.20)  0.09 (0.03 – 0.25) | 0.34 | 0.88 (0.72 – 1.08)  1.16 (0.89 – 2.41) | 0.15 | 0.45 (0.38 – 0.53)  0.63 (0.35 – 1.15) | 0.25 |
| HBV coinfection  No  Yes | 0.14 (0.10 – 0.19)  0.20 (0.07 – 0.56) | 0.59 | 0.91 (0.75 – 1.11)  1.11 (0.50 – 2.45) | 0.68 | 0.46 (0.39 – 0.54)  0.59 (0.20 – 1.71) | 0.54 |
| HCV coinfection  No  Yes | 0.14 (0.10 – 0.20)  0.11 (0.03 – 0.45) | 0.66 | 0.93 (0.77 – 1.14)  0.75 (0.29 – 1.94) | 0.68 | 0.48 (0.40 – 0.56)  0.37 (0.21 – 0.65) | 0.55 |

**^*^** Back-transformed log values

**Table S3**

**Correlation between persistent organic pollutants concentrations and characteristics (as continuous values) of patients**

| **Characteristic** | **Chlordecone** | | **DDE** | | **PCB-153** | |
| --- | --- | --- | --- | --- | --- | --- |
|  | **r *** | ***P*** | **r *** | ***P*** | **r *** | ***P*** |
| Age at enrolment | 0.014 | 0.85 | 0.427 | <0.0001 | 0.460 | <0.0001 |
| Total plasma lipids | -0.099 | 0.19 | 0.103 | 0.17 | 0.149 | 0.05 |
| Chlordecone |  |  | 0.003 | 0.97 | 0.115 | 0.13 |
| DDE |  |  |  |  | 0.486 | <0.0001 |

**^*^** Pearson correlation coefficient

**Table S4**

**Univariate analysis of risk factors for the progression of liver fibrosis**

| **Characteristics** | **No fibrosis (F0)**  **N (%)** | **Cirrhosis (F4)**  **N (%)** | **HR** | ***P*** |
| --- | --- | --- | --- | --- |
| Age at enrolment, years (mean, SD) | 49.5 (9.3) | 55.3 (11.5) | 0.89 (0.86 – 0.92) | <0.0001 |
| Sex  Men  Women | 41 (91.1)  4 (8.9) | 97 (86.6)  15 (13.4) | 1.00  1.09 (0.54 – 2.18) | 0.82 |
| Education  Primary  Secondary  High School and higher | 8 (17.8)  30 (66.7)  7 (15.6) | 29 (25.9)  72 (64.3)  11 (9.8) | 1.00  2.32 (1.36 – 3.95)  3.22 (1.43 – 7.27) | 0.002  0.005 |
| Body Mass Index, kg/m^2^  Normal (< 25)  Overweight (25 - <30)  Obesity (>30) | 39 (86.7)  5 (11.1)  1 (2.2) | 79 (70.5)  29 (25.9)  4 (3.6) | 1.00  1.16 (0.71 – 1.89)  1.10 (0.34 – 3.53) | 0.56  0.87 |
| Weight loss > 10 % during the last 10 years  No  Yes | 7 (15.6)  38 (84.4) | 24 (21.4)  88 (78.6) | 1.00  1.21 (0.74 – 1.98) | 0.45 |
| Age at alcohol onset, years  < 40  > 40 | 40 (88.9)  5 (11.1) | 104 (92.9)  8 (7.1) | 1.00  20.8 (8.00 – 54.1) | <0.0001 |
| Smoking  Never  Ever | 19 (42.2)  26 (57.8) | 61 (54.5)  51 (45.5) | 1.00  1.61 (1.04 – 2.48) | 0.03 |
| Coffee consumption  Never  Ever | 27 (60.0)  18 (40.0) | 62 (55.4)  50 (44.6) | 1.00  0.67 (0.43 – 1.02) | 0.06 |
| Recreational drugs  Never  Ever | 41 (91.1)  4 (8.9) | 105 (93.8)  7 (6.3) | 1.00  1.90 (0.96 - 4.17) | 0.11 |
| Diabetes Mellitus  No  Yes | 43 (95.6)  2 (4.4) | 96 (85.7)  16 (14.3) | 1.00  1.97 (1.01 – 3.86) | 0.05 |
| HBV coinfection  No  Yes | 42 (93.3)  3 (6.7) | 109 (97.3)  3 (2.7) | 1.00  0.53 (0.13 – 2.15) | 0.37 |
| HCV coinfection, n (%)  No  Yes | 42 (93.3)  3 (6.7) | 106 (94.6)  6 (5.4) | 1.00  0.71 (0.26 – 1.95) | 0.51 |
| Total plasma lipids, g/L (mean, SD) | 4.58 (1.37) | 4.78 (1.54) | 0.95 (0,85 – 1,06) | 0.35 |

**Table S5**

**Plasma albumin and total and conjugated bilirubin concentrations according to the stage of liver fibrosis**

| **Variable** | **Mean * (CI 95 %)** | ***P*** |
| --- | --- | --- |
| Albumin (g/l)  No fibrosis (F0)  Cirrhosis (F4) | 37.8 (35.8 – 39.7)  30.2 (28.7 – 31.8) |  |
|  |  | <0.001 |
| Total bilirubin (µmol/l)  No fibrosis (F0)  Cirrhosis (F4) | 16.0 (13.1 – 19.4)  42.5 (34.3 – 52.7) |  |
|  |  | <0.001 |
| Conjugated bilirubin (µmol/l)  No fibrosis (F0)  Cirrhosis (F4) | 9.7 (7.6 – 12.4)  40.4 (31.7– 51.5) |  |
|  |  | <0.001 |

**^*^** Back-transformed log values for total and conjugated bilirubin
